# Pollutants in Microenvironmental Cellular Interactions During Liver Inflammation Cancer Transition and the Application of Multi-Omics Analysis

**DOI:** 10.3390/toxics13030163

**Published:** 2025-02-25

**Authors:** Yulun Jian, Yuhan Li, Yanfeng Zhou, Wei Mu

**Affiliations:** School of Public Health, Center for Single-Cell Omics, Shanghai Jiao Tong University School of Medicine, Shanghai 200025, China; 1418398652@sjtu.edu.cn (Y.J.); 123711910099@sjtu.edu.cn (Y.L.); yfzhou@shsmu.edu.cn (Y.Z.)

**Keywords:** inflammatory cancer transformation, liver microenvironment, cellular interaction, contaminants, exposomics

## Abstract

This study categorizes pollutant-induced inflammation–cancer transition into three stages: non-alcoholic fatty liver disease (NAFLD), liver fibrosis, and hepatocellular carcinoma (HCC). It systematically reveals the temporal heterogeneity of pollutant-induced liver damage. The findings indicate that pollutants not only directly damage hepatocytes but also modulate key cells in the immune microenvironment, such as hepatic stellate cells (HSCs) and Kupffer cells, thereby amplifying inflammatory and fibrotic responses, ultimately accelerating the progression of HCC. Mechanistically, in the early stage (NAFLD), pollutants primarily cause hepatocyte injury through oxidative stress and lipid metabolism dysregulation. During the fibrosis stage, pollutants promote liver fibrosis by inducing extracellular matrix accumulation, while in the HCC stage, they drive tumorigenesis via activation of the Wnt/β-catenin pathway and p53 inactivation. Through multi-omics analyses, this study identifies critical pathogenic molecules and signaling pathways regulated by pollutants, providing new insights into their pathogenic mechanisms, potential biomarkers, and therapeutic targets. These findings offer valuable guidance for the development of diagnostic and therapeutic strategies for liver diseases and the formulation of environmental health risk prevention measures.

## 1. Introduction

**Figure 1:**
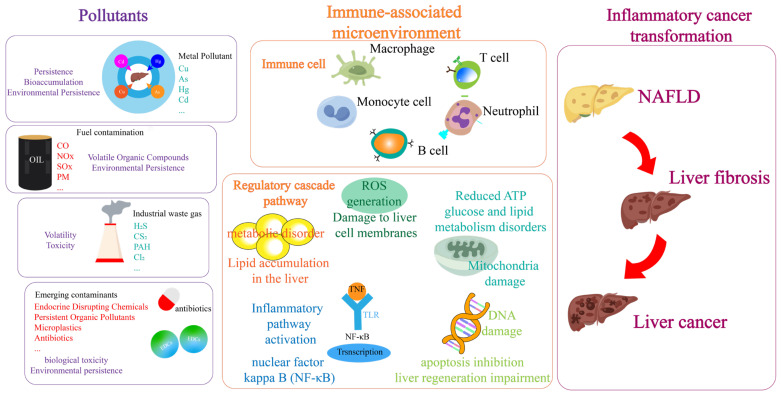
A schematic representation of possible mechanism underlying hepatotoxicity. Various environmental pollutants, including metal contaminants, fossil fuel combustion by-products, and persistent organic pollutants, induce the pathological development of liver diseases through prolonged exposure, involving multiple biological levels. At the cellular level, pollutants contribute to liver disease not only by disrupting immune cells within the liver, such as macrophages and B cells, but also by recruiting extrahepatic immune cells that affect the immune microenvironment. In terms of pathway regulation, pollutants impair critical signaling pathways by reducing ATP production, causing mitochondrial metabolic dysfunction, and activating the NF-κB pathway to interfere with cytokine release. This disruption triggers hepatic lesions, beginning with liver steatosis (non-alcoholic fatty liver), progressing to fibrosis and tissue remodeling, and ultimately potentially leading to liver cancer. This gradual progression underscores the crucial role of long-term exposure in the pollution-mediated development of liver disease.

Chemical-induced hepatotoxicity represents significant global environmental and health concerns, contributing to progressive liver dysfunction and irreversible structural alterations [1,2]. With increasing environmental pollution, the liver disease spectrum typically represents a complex, multi-stage pathological process characterized by the progression from chronic inflammation and NAFLD to fibrosis and ultimately hepatocellular carcinoma (HCC) [3]. Chronic pollutant exposure induces oxidative stress, triggering apoptotic pathways and hepatic stellate cell activation [4], while persistent inflammation establishes a pro-carcinogenic microenvironment [5]. Concurrent DNA damage and epigenetic modifications promote hepatocarcinogenesis [6], followed by activation of angiogenic factors facilitating tumor progression [7]. Although extensive research has examined the molecular mechanisms of pollutant-induced liver injury, most studies emphasize acute exposure models [8,9], whereas environmental scenarios typically involve chronic, low-dose exposures. Current investigations have not fully elucidated the complex mechanisms by which persistent pollutant retention modulates the hepatic microenvironment, facilitating the inflammation-cancer transition. Therefore, there is an urgent need to delineate the molecular signaling cascades mediating pollutanthepatocyte interactions and to develop advanced technologies for identifying and validating novel biomarkers associated with pollutant-induced pathogenesis and therapeutic interventions. This comprehensive understanding would provide crucial insights into both diagnostic strategies and targeted therapeutic approaches (Figure 1).

## 2. The Impact of Pollutants on the Entire Cycle of Disease Stages of Liver Injury Leading to Hepatocellular Carcinoma

The liver, a xenobiotic metabolic center and major immune organ, harbors diverse immune cells, including Kupffer cells, NK cells, NKT cells, and lymphocytes [10]. These immune cells interact closely with hepatocytes within the hepatic microenvironment to maintain homeostasis and respond to pathogens or toxins. Under physiological conditions, Kupffer cells maintain immune tolerance by secreting anti-inflammatory cytokines (TNF-α, IL-6). NK cells and NKT cells monitor and eliminate abnormal hepatocytes. However, when the liver experiences sustained damage, it triggers the process of inflammation cancer transformation. In the early stages of chronic liver injury, damaged hepatocytes release damage-associated molecular patterns (DAMPs), which activate Kupffer cells. This activation induces a phenotypic shift in Kupffer cells from the M2 to the M1 phenotype, resulting in the secretion of pro-inflammatory cytokines such as TNF-α, IL-6, and IL-1β. These cytokines not only promote hepatocyte injury but also activate HSCs, initiating fibrosis. Concurrently, the chronic inflammatory environment induces hepatocytes to express PD-L1, inhibiting the anti-tumor activity of CD8+ T cells and creating conditions for immune evasion. Persistent inflammatory stimuli lead to immune cell dysfunction, with impaired cytotoxic functions in NK and NKT cells, as evidenced by the upregulation of inhibitory receptors such as NKG2A. In the later stages of inflammation cancer transformation, tumor-associated macrophages (TAMs) accumulate significantly in the tumor microenvironment, predominantly exhibiting an M2 phenotype. These TAMs secrete factors like TGF-β and VEGF, promoting angiogenesis and immune suppression. Hepatic stellate cells are transformed into cancer-associated fibroblasts (CAFs), which secrete extracellular matrix components and growth factors to facilitate tumor growth [11]. During this inflammation cancer transformation, environmental pollutants can initiate and promote liver damage through multiple mechanisms. Environmental and foodborne pollutants can directly cause cytotoxicity in hepatic parenchymal cells [12] or regulate cell cell communications and cell matrix interactions, all of which trigger sophisticated molecular cascade effects [13]. Pollutants can directly target hepatocytes, causing oxidative stress, mitochondrial dysfunction, and DNA damage, which may lead to cellular death or malignant transformation [14]. Moreover, these toxicants can disrupt the intricate crosstalk between different hepatic cell populations (including hepatocytes, Kupffer cells, hepatic stellate cells, and sinusoidal endothelial cells), thereby activating inflammatory responses and promoting fibrogenic pathways [15]. The resulting molecular cascades, including cytokine signaling, growth factor activation, and matrix remodeling, drive a pro-inflammatory and pro-fibrotic microenvironment that exacerbates liver damage and fosters hepatocarcinogenesis [16]. However, current research primarily focuses on the effects of pollutants on individual liver cell types or specific stages of disease while largely ignoring the molecular mechanisms underlying intercellular interactions. Moreover, there is a significant lack of comprehensive elucidation regarding the key target cells and their toxic molecular pathways during disease progression.

### 2.1. Pollutant-Induced Non-Alcoholic Fatty Liver Disease (NAFLD)

Non-alcoholic fatty liver disease (NAFLD) is a metabolic disorder characterized by excessive lipid accumulation in hepatocytes and is closely associated with obesity, insulin resistance, and dyslipidemia [17]. The pathogenesis of NAFLD is driven by intricate intercellular interactions among hepatocytes, Kupffer cells, hepatic stellate cells (HSCs), and infiltrating immune cells, which are regulated by a complex network of signaling pathways. Environmental pollutants have recently been identified as significant contributors to the progression of NAFLD. These pollutants disrupt hepatocellular function and intercellular communication, either directly or indirectly, thereby accelerating disease progression [18].

At the hepatocellular level, environmental pollutants serve as initiators of NAFLD by disrupting lipid metabolism and inducing cellular damage [19]. Exposure to persistent organic pollutants (POPs) and plasticizers perturbs lipid homeostasis by upregulating lipogenic pathways, such as sterol regulatory element-binding protein-1c (SREBP-1c) and peroxisome proliferator-activated receptor γ (PPAR-γ), while concurrently suppressing fatty acid β-oxidation [20]. These effects drive excessive lipid accumulation in hepatocytes. In addition, pollutants activate the aryl hydrocarbon receptor (AhR) signaling pathway, leading to the overexpression of metabolic enzymes such as CYP1A1, which exacerbates mitochondrial dysfunction and promotes the generation of reactive oxygen species (ROS) [21]. The resultant oxidative stress and mitochondrial damage trigger hepatocyte apoptosis and the release of damage-associated molecular patterns (DAMPs), such as HMGB1 and extracellular ATP. These DAMPs subsequently activate immune cells, initiating local inflammation and amplifying tissue damage. Kupffer cells, the liver-resident macrophages, are key mediators of pollutant-induced inflammation. Pollutants activate pattern recognition receptors (PRRs), such as Toll-like receptors (TLRs) and NOD-like receptors (NLRs), in Kupffer cells, thereby stimulating downstream signaling pathways, including NF-κB and MAPK [22]. This activation enhances the production of pro-inflammatory cytokines, such as TNF-α, IL-1β, and IL-6, as well as chemokines, including CCL2. These inflammatory mediators not only amplify hepatocyte injury but also recruit peripheral immune cells, such as monocytes and neutrophils, to the liver [23]. The infiltrating immune cells, in turn, release additional inflammatory mediators, reactive oxygen species, and proteolytic enzymes, further exacerbating tissue inflammation and hepatocyte damage. This creates a self-sustaining cycle of inflammation and injury that accelerates disease progression.

The activation of hepatic stellate cells (HSCs) represents a pivotal step in the progression of NAFLD-associated fibrosis, and environmental pollutants significantly contribute to this process [24]. DAMPs released by injured hepatocytes, along with pro-fibrotic cytokines such as TGF-β and PDGF secreted by activated Kupffer cells, synergistically induce the HSC activation. Once activated, HSCs adopt a myofibroblast-like phenotype, characterized by excessive production of extracellular matrix (ECM) proteins and activation of pro-fibrotic pathways, such as the TGF-β/Smad signaling cascade. Furthermore, activated HSCs modulate the hepatic microenvironment through paracrine signaling, sustaining immune cell activation and altering ECM composition, which collectively impede hepatocyte regeneration and repair, thereby worsening liver fibrosis [25].

The effects of environmental pollutants are not confined to individual cell types but extend to the disruption and reprogramming of intercellular communication networks within the liver [26]. For instance, pollutant-induced activation of the AhR pathway not only exacerbates metabolic dysfunction in hepatocytes but also modulates the inflammatory and fibrotic responses of Kupffer cells and HSCs. These multi-level interactions contribute to the establishment of a pro-inflammatory and pro-fibrotic microenvironment, fostering the progression of NAFLD [27].

In conclusion, NAFLD is not a result of isolated cellular events but is driven by complex intercellular interactions involving hepatocytes, Kupffer cells, hepatic stellate cells, and infiltrating immune cells. Environmental pollutants significantly exacerbate NAFLD pathogenesis by directly or indirectly altering the biological behavior of these cells, thereby reshaping the hepatic cellular communication network. These pollutant-induced intercellular interactions promote inflammation while simultaneously impairing the liver’s regenerative capacity, ultimately accelerating the progression of NAFLD toward irreversible liver injury. A comprehensive understanding of these mechanisms is essential for the development of targeted therapeutic strategies to mitigate pollutant-induced NAFLD.

### 2.2. Pollutant Exposure in the Regulation of Disease Progression from Liver Fibrosis to Cirrhosis

Hepatic fibrosis is a pathological process triggered by chronic liver injury, characterized by the abnormal deposition of extracellular matrix (ECM) proteins, particularly collagen [28]. When the progression of hepatic fibrosis surpasses the liver’s reparative capacity, it eventually leads to the development of cirrhosis, an irreversible end-stage liver disease. Cirrhosis is marked by the destruction of hepatic lobular architecture, the formation of abnormal fibrous septa, and the development of regenerative nodules, accompanied by significant hepatic dysfunction and portal hypertension [29]. The transition from hepatic fibrosis to cirrhosis is primarily driven by persistent chronic inflammation, excessive activation of hepatic stellate cells (HSCs), and remodeling of the hepatic microenvironment.

During this pathological progression, different types of environmental pollutants exacerbate liver injury and fibrosis progression through multiple mechanisms. In the early stages of fibrosis, pollutants interfere with hepatic lipid metabolism and mitochondrial function, leading to the accumulation of ROS and activation of pro-inflammatory signaling pathways. This induces lipid peroxidation, damages mitochondrial DNA, and activates cellular stress responses, further exacerbating lipotoxicity and promoting the fibrotic cascade, which directly leads to hepatocyte death and the release of damage-associated molecular patterns (DAMPs), such as HMGB1 and extracellular ATP. These DAMPs activate intrahepatic immune cells, including Kupffer cells, which secrete pro-inflammatory cytokines (e.g., TNF-α, IL-6) and chemokines (e.g., CCL2) to recruit peripheral immune cells, such as monocytes and neutrophils, to the liver, further amplifying the inflammatory response [30].

The synergistic effects of heavy metals and organic pollutants play a key role in this process. Heavy metals (e.g., cadmium, lead) deplete cellular glutathione (GSH) reserves and inhibit antioxidant enzyme activity, while organic pollutants (e.g., PAHs) generate large amounts of reactive oxygen species (ROS) through the cytochrome P450 (CYP450) metabolic system [31]. This dual insult not only severely impairs mitochondrial function but also activates the MAPK and NF-κB signaling pathways, which synergize with pollutant-induced pro-inflammatory factor expression via the AhR signaling pathway, thereby exacerbating the inflammatory response [32].

Endocrine-disrupting chemicals (EDCs) and other environmental pollutants exacerbate liver injury primarily by interfering with metabolic homeostasis. EDCs disrupt lipid metabolism through nuclear receptors (e.g., PPARs, LXR) and, in conjunction with other pollutants that impair glucose metabolism, lead to more severe lipid accumulation and insulin resistance. Moreover, pollutants can directly stimulate Kupffer cells by activating aryl hydrocarbon receptors (AhRs) and pattern recognition receptors (PRRs), such as TLRs and NLRs, leading to the secretion of large amounts of pro-inflammatory and pro-fibrotic factors.

During fibrosis progression, DAMPs released by damaged hepatocytes and pro-fibrotic signals (e.g., TGF-β and PDGF) secreted by activated Kupffer cells synergistically induce the activation of HSCs. Activated HSCs are characterized by the excessive secretion of ECM proteins (e.g., type I and III collagen) and the upregulation of fibrotic signaling pathways, such as the TGF-β/Smad and Wnt/β-catenin pathways. Pollutants further promote fibrosis through pathways such as TGF-β/Smad and PI3K/Akt, upregulating the expression of fibrosis-related genes. In addition, pollutant exposure alters the hepatic microenvironment by inhibiting the activity of matrix metalloproteinases (MMPs) and upregulating their inhibitors (TIMPs), thereby reducing ECM degradation and exacerbating fibrosis [33].

Pollutants also show significant synergistic effects in exacerbating viral hepatitis. Pollutants can alter the epigenetic status of the viral genome, such as by modifying DNA methylation patterns, potentially enhancing viral replication. Additionally, pollutants suppress the immune system, reducing antiviral immune surveillance and leading to chronic viral infection and aggravated liver injury. At the molecular level, viral infection activates PRRs (e.g., TLR3/RIG-I), which synergize with pollutant-stimulated PRRs (e.g., TLR4) to amplify the inflammatory response [34]. Recent studies have demonstrated that non-coding RNAs play critical roles in pollutant-induced hepatic fibrosis. Environmental pollutants can alter the expression profiles of multiple microRNAs, influencing the expression of fibrosis-related genes. Simultaneously, pollutant-induced changes in long non-coding RNA (lncRNA) expression can enhance HSC activation through epigenetic modifications. Furthermore, pollutants can induce ferroptosis by disrupting iron metabolism and promoting lipid peroxidation or induce pyroptosis by activating the NLRP3 inflammasome. These newly identified forms of cell death provide novel insights into the toxic mechanisms of pollutants [35].

In summary, the transition from hepatic fibrosis to cirrhosis is a pathological process driven by the synergistic effects of various environmental pollutants through complex molecular networks. Understanding the molecular mechanisms underlying these synergistic effects not only sheds light on the role of environmental factors in liver disease progression but also provides an important theoretical basis for developing novel therapeutic strategies. Future research should focus on the integration of multi-omics data and the development of advanced research models to elucidate the complex mechanisms of pollutant-induced hepatic fibrosis from a systems biology perspective.

### 2.3. Environmental Pollutants Promote HCC Progression and Metastasis

The International Agency for Research on Cancer (IARC) and the World Health Organization (WHO) confirmed that prolonged exposure to carcinogenic pollutants significantly increases the risk of hepatocellular carcinoma (HCC) [36]. As the terminal stage of chronic liver disease, HCC typically evolves from fibrosis and cirrhosis and represents one of the most critical endpoints of environmental pollutant exposure. Traditionally, it has been recognized that carcinogenic pollutants promote HCC initiation and metastasis by disrupting the normal physiological processes of hepatocytes and altering the hepatic microenvironment. For instance, dioxins and polycyclic aromatic hydrocarbons (PAHs) increase HCC risk by inducing DNA damage and inhibiting DNA repair mechanisms, while polychlorinated biphenyls (PCBs) accelerate HCC progression by promoting hepatocyte proliferation and interfering with cell cycle regulation [37]. Notably, our previous research demonstrated that low-dose exposure to dietary benzo[a]pyrene, although insufficient to directly induce tumor cell proliferation, significantly enhances the metastatic potential of HCC cells, particularly metastasis to the lungs [36]. Clinically, metastasis is a major factor contributing to the reduced five-year survival rate of HCC patients. These findings underscore the importance of dietary management and clinical nutrition in the care of cancer patients.

#### 2.3.1. Interactions Between Tumor Cells and Stromal Cells

The invasive and metastatic characteristics of HCC, especially its tropism for lung metastasis, are closely associated with the tumor microenvironment (TME). Whether environmental pollutants influence the interactions between host cells and tumor cells within the TME has become a critical research focus. Recent studies have revealed that pollutants regulate HCC metastasis through exosome-mediated mechanisms. For example, exposure to benzo[a]pyrene induces HCC cells to release exosomal circular RNAs (circRNAs), which promote organ-specific metastasis, particularly to the lungs. These exosomal circRNAs are taken up by pulmonary fibroblasts, where they enhance cell proliferation and fibrosis, modulate immune cell activity within the lung microenvironment, suppress local anti-tumor immune responses, and ultimately enhance lung metastasis. Pollutant-induced exosome production not only alters the hepatic microenvironment but also affects the distant organ microenvironment, facilitating tumor cell colonization and metastatic potential. Additionally, increasing evidence suggests that environmental pollutants drive HCC progression by altering the activation state of hepatic stellate cells (HSCs) through tumor cell-mediated interactions. These interactions not only reshape the TME but also exacerbate liver fibrosis and tumor progression. Pollutants regulate tumor cell HSC interactions by activating key molecular signaling pathways, including NF-κB, STAT3, TGF-β/Smad, and Hedgehog signaling. For example, the TGF-β/Smad pathway is activated in HSCs by TGF-β secreted from tumor cells, promoting collagen synthesis and hepatic fibrosis. Similarly, the NF-κB and STAT3 pathways are activated by pro-inflammatory cytokines (e.g., IL-6 and IL-1β) secreted by tumor cells, driving pro-fibrotic and immunosuppressive effects.

#### 2.3.2. Interactions Between Tumor Cells and Immune Cells

Environmental pollutants also modulate the various cellular components of the immune microenvironment in HCC. Macrophages in the hepatic TME exhibit two distinct polarization states: M1 macrophages exhibit anti-tumor properties by producing pro-inflammatory cytokines such as IL-12 and TNF-α, whereas M2 macrophages promote tumor progression by secreting anti-inflammatory cytokines such as IL-10 and TGF-β and enhancing angiogenesis. Persistent organic pollutants (e.g., PCBs and dioxins) have been shown to induce M2 macrophage polarization, thereby enhancing the pro-tumor microenvironment [38]. In addition to macrophages, other immune cells within the TME are also affected by pollutant exposure. Cytotoxic CD8+ T cells, which play a critical role in anti-tumor immunity, are suppressed by pollutants such as benzo[a]pyrene and dioxins. These pollutants induce oxidative stress in CD8+ T cells, triggering inflammatory signaling pathways. This oxidative stress results in reduced T-cell receptor expression, diminished cytokine production such as IFN-γ, and impaired T-cell activation. Unlike the generalized oxidative stress observed in hepatocytes, the process in T cells specifically interferes with their immune functions, contributing to the suppression of anti-tumor responses. Furthermore, the interaction between ALDOB and KAT2A has been shown to epigenetically regulate TGF-β expression, further impairing T-cell function and promoting HCC progression [34].

**Figure 2:**
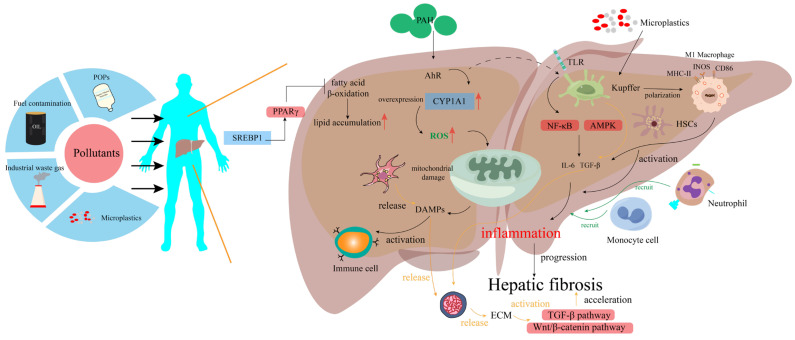
Illustration of cell crosstalk and molecular pathways of different chemicals in the liver microenvironment. Pollutants such as persistent organic pollutants (POPs) and microplastics, upon reaching the liver through inhalation or dietary intake, trigger complex cellular interactions. POPs upregulate lipid biosynthesis pathways such as SREBP-1c and PPAR-γ, disrupting lipid homeostasis, inhibiting fatty acid β-oxidation, and causing excessive lipid accumulation in hepatocytes. Pollutants also activate the AhR signaling pathway, leading to the overexpression of metabolic enzymes like CYP1A1, exacerbating mitochondrial dysfunction and promoting ROS generation. The resulting oxidative stress and mitochondrial damage initiate hepatocyte apoptosis and the release of DAMPs, such as HMGB1 and extracellular ATP. These DAMPs subsequently activate immune cells, amplifying tissue damage. Through the activation of TLEs and NLRs in Kupffer cells, downstream signaling pathways, including NF-κB and MAPK, are stimulated, enhancing pro-inflammatory cytokine production. Inflammatory mediators recruit peripheral immune cells such as monocytes and neutrophils to the liver, further aggravating tissue inflammation and hepatocyte injury. DAMPs released by damaged hepatocytes, in conjunction with pro-fibrotic cytokines secreted by activated Kupffer cells, synergistically induce the activation of hematopoietic stem cells, leading to excessive ECM protein production and activation of the TGF-β/Smad signaling cascade. This impedes hepatocyte regeneration and repair, exacerbating liver fibrosis. The interactions between hepatic parenchymal cells and immune cells drive inflammation, metabolic signaling networks, and immune stress responses.

B cells within the TME exhibit dual roles: they can produce anti-tumor antibodies or secrete pro-inflammatory factors that support tumor growth. Pollutants such as cadmium and arsenic have been reported to promote the differentiation of B cells into phenotypes that secrete IL-10, thereby fostering a tumor-promoting microenvironment [39]. Natural killer (NK) cells, which are essential for innate anti-tumor immunity, are also negatively impacted by environmental pollutants. For instance, PCBs downregulate the expression of activating receptors (e.g., NKG2D) on NK cells, impairing their ability to recognize and target tumor cells. Similarly, exposure to PM2.5 promotes the production of immunosuppressive cytokines (e.g., IL-10 and TGF-β), significantly reducing the cytotoxic activity of NK cells [40]. These findings deepen our understanding of the carcinogenic mechanisms of environmental pollutants and provide a foundation for the development of novel therapeutic strategies targeting tumor cell–stromal cell interactions (Figure 2).

## 3. Mechanistic Studies of Chemical Exposomics and Liver Injury

Chemical-induced carcinogenesis involves various biological changes, including gene mutations, epigenetic regulation, transcriptional alterations, protein dysfunction, and metabolic disruptions. Environmental carcinogenic risk factors such as silica, tobacco smoke, arsenic, dust, and PM2.5 have been shown to induce the expression of the Mineral Dust-Induced Gene (MDIG) in multiple cell types. The MDIG is considered an epigenetic regulator involved in histone demethylation, a process observed during tumor progression [41]. Additionally, exposure to the chemical carcinogen diethylnitrosamine (DEN) promotes the production of IL-6 in Kupffer cells through an MyD88-dependent pathway, potentially contributing to the development of HCC [42]. Despite substantial progress in elucidating the role of environmental pollutants in HCC initiation and progression, many aspects of the specific molecular targets and core mechanisms underlying chemical carcinogenesis remain unclear. Since these complex mechanisms cannot be fully elucidated using single-method approaches, integrative multi-omics research, including genomics, transcriptomics, proteomics, metabolomics, and epigenomics, has emerged as a critical direction for investigating the mechanisms of chemical carcinogenesis. By combining high-throughput technologies with bioinformatics analysis tools, multi-omics approaches enable the identification of key targets and biomarkers associated with chemical carcinogenesis.

Chemical exposomics is an emerging field aimed at systematically studying the total lifetime exposure of an individual to external chemical agents, collectively referred to as the “exposome”, and its biological impact on health [43]. Unlike traditional environmental studies that focus on single pollutants, exposomics seeks to capture the entirety of human exposure to environmental chemicals (e.g., pollutants in air, water, food, or soil) and lifestyle factors (e.g., diet, medications, smoking). By integrating multi-omics technologies such as genomics, transcriptomics, proteomics, and metabolomics, exposomics provides a comprehensive framework to explore the interplay between environmental exposures and human health or disease. Exposomics lies in the comprehensive characterization of external chemical exposures and their metabolic transformations within the body. Using high-throughput analysis techniques like mass spectrometry (MS) and nuclear magnetic resonance (NMR), exposomics quantifies chemical components in biological samples, evaluates their temporal and dose-dependent dynamics, and identifies complex exposure health associations using advanced data integration and bioinformatics tools. The ultimate goal is to construct an individual “exposure map” or exposome, which encompasses all exposures from birth to death, integrating these data with genomic information to elucidate how environmental and genetic factors collectively contribute to disease onset and progression [44].

Exposomics provides unprecedented insights into the mechanisms of chemical pollutant-induced toxicity by integrating multi-omics approaches. This integration facilitates a holistic analysis of pollutant effects across multiple biological levels, spanning genomics, transcriptomics, proteomics, and metabolomics. Such approaches not only enhance our understanding of liver cancer pathogenesis but also clarify the intricate relationships between environmental pollutants and liver pathology. For instance, exposomics has revealed how exogenous chemical exposures modulate gene expression, protein modifications, and metabolic pathways during liver injury and carcinogenesis. The systematic integration of multi-omics data offers a comprehensive view of the mechanisms underlying pollutant-induced liver injury, enabling the identification of novel biomarkers and therapeutic targets.

### 3.1. Genomics and Transcriptomics in Pollutant-Induced Liver Injury

Advancements in genomics and transcriptomics offer powerful tools for elucidating the mechanisms underlying pollutant-induced liver injury. Genomics provides unique insights at the DNA level, revealing the toxic mechanisms of chemical exposure. Whole-genome sequencing and DNA damage detection techniques allow systematic assessment of pollutant-induced genomic alterations, including the extent and type of DNA damage. For instance, environmental toxins such as dioxins and polycyclic aromatic hydrocarbons (PAHs) have been shown to cause DNA double-strand breaks, base modifications, and chromosomal abnormalities, leading to increased genomic instability. Without timely repair, this DNA damage may result in cellular dysfunction, hepatocyte death, or carcinogenesis [45]. Next-generation sequencing has enabled the identification of specific mutation patterns associated with chemical exposures, which serve as molecular fingerprints for particular pollutants. Such findings establish causal relationships between chemical exposure and liver injury, offering new perspectives for environmental carcinogen screening and risk assessment. Chromosomal alterations, including large-scale rearrangements such as breaks, translocations, and copy number variations, have been implicated in severe liver diseases, particularly during chronic chemical exposure leading to liver cancer. Genomic studies also reveal that pollutant exposure can impair DNA repair pathways by downregulating the expression or function of DNA repair genes, further exacerbating genomic instability. This comprehensive genomic analysis not only deepens our understanding of pollutant toxicity but also provides valuable references for biomarker discovery and intervention strategies [46].

Transcriptomics complements genomics by uncovering how pollutants regulate gene expression. Transcriptomic approaches enable the detection of dynamic changes in gene expression following pollutant exposure, thereby identifying key genes and signaling pathways involved in liver injury. For example, dioxin exposure has been shown to upregulate pro-inflammatory cytokine genes such as TNF-α, IL-6, and IL-1β, while disrupting the expression of metabolic pathway genes, such as cytochrome P450 family members [47]. In mouse experiments, TCDD exposure triggered changes in metabolites and gene expression related to lipid metabolism and transport, choline metabolism, bile acid metabolism, glycolysis, and glycerophospholipid metabolism. In C57 mice, alterations in lipid metabolism led to increased hepatic triacylglycerol levels, as well as elevated levels of monounsaturated fatty acids (FAs) [48]. These findings highlight the pivotal roles of inflammation and metabolic imbalance in dioxin-induced liver injury. Transcriptomics also provides insights into dose response and time-dependent changes in gene expression, offering valuable data for toxicity assessment and safe exposure limits.

Pollutants not only affect mRNA expression but also influence RNA epigenetic modifications, such as N6-methyladenosine (m6A) methylation, which regulates RNA stability, splicing, degradation, and translation. Transcriptomic studies have found that pollutants like PAHs and heavy metals significantly alter the m6A methylation landscape in hepatocytes, affecting the translation efficiency of key genes. For instance, arsenic exposure increases m6A modifications on oxidative stress-related genes, enhancing mRNA stability and exacerbating oxidative stress and liver damage [49]. In mouse models, exposure to the heavy metal cadmium interferes with the expression levels of the m6A methyltransferase METTL3, leading to methylation dysregulation and resulting in liver damage [50]. High-throughput sequencing of RNA epigenetic modifications provides novel perspectives on the molecular mechanisms of pollutant toxicity. Additionally, transcriptomics has made significant progress in understanding the roles of non-coding RNAs in pollutant-induced liver injury. Long non-coding RNAs (lncRNAs) and microRNAs (miRNAs) are key regulators of gene expression and have been implicated in pollutant-induced liver damage [51]. For example, specific lncRNAs are dysregulated in the liver following pollutant exposure and may modulate gene expression by interacting with transcription factors or mRNAs. Meanwhile, changes in miRNA expression can influence inflammation and fibrosis by targeting mRNAs for degradation or translation inhibition. Some lncRNAs and miRNAs have been linked to liver fibrosis and hepatocarcinogenesis. For example, after sustained exposure to the heavy metal cadmium in cows, transcriptomic analysis of the liver identified 24 differentially expressed miRNAs and 169 differentially expressed lncRNAs. Differentially expressed miRNAs (DEmiRNAs) included bta-miR-12051, bta-miR-211, bta-miR-222, and bta-miR-11986c. Differentially expressed lncRNAs (DElncRNAs) included 107,132,706, 104,972,497, 790,183, and 104,973,640, which are particularly important in the regulation of liver injury. These findings suggest the potential of these RNA molecules as biomarkers for pollutant exposure.

By integrating genomic and transcriptomic data, researchers can comprehensively analyze the mechanisms of pollutant toxicity, from DNA damage to gene expression regulation. For example, in studies of dioxin-induced liver injury, genomic analyses have identified DNA damage and specific mutation patterns, while transcriptomic studies have highlighted the dysregulation of genes involved in inflammation, metabolic imbalance, and cell death. Integrated analyses have further uncovered the activation of the aryl hydrocarbon receptor (AhR) signaling pathway as a central molecular event. Similarly, in studies of heavy metal exposure (e.g., cadmium and arsenic), combined genomic and transcriptomic analyses have revealed characteristic DNA damage patterns and oxidative stress-related gene expression changes, elucidating the full toxicological chain from genomic damage to transcriptional regulation. This integrated approach provides a comprehensive understanding of pollutant-induced liver injury mechanisms and lays a solid foundation for pollutant risk evaluation and intervention strategies.

### 3.2. Proteomics and Metabolomics in Pollutant-Induced Liver Injury

Proteomics focuses on the comprehensive study of protein composition, expression levels, post-translational modifications, interactions, and functions within cells, tissues, or organisms. It has become a powerful tool for elucidating the molecular mechanisms of pollutant-induced liver injury [52]. Proteins, as direct executors of cellular functions, are often profoundly affected by pollutants, and their alterations reflect the severity of liver toxicity. Pollutants frequently disrupt post-translational modifications (PTMs), such as phosphorylation and acetylation, altering protein activity, stability, localization, and interactions. For example, PCBs significantly alter the phosphorylation levels of proteins involved in signal transduction, cell cycle regulation, and metabolism [53]. Heavy metals such as cadmium and arsenic interfere with acetylation processes, disrupting energy metabolism and oxidative stress responses in hepatocytes [54]. These PTM changes serve as both markers of liver damage and key mechanisms of pollutant toxicity. Mass spectrometry-based proteomics has become a central analytical tool for studying pollutant-induced liver injury. This approach relies on liquid chromatography tandem mass spectrometry (LC-MS/MS) systems, using data-dependent acquisition (DDA) and data-independent acquisition (DIA) modes to obtain protein information. Techniques such as isotopic labeling (e.g., TMT, iTRAQ) or label-free quantification enable precise protein quantification. In PTM analysis, enrichment methods such as IMAC (immobilized metal affinity chromatography) and TiO2-based methods, combined with high-resolution mass spectrometry, allow the identification and quantification of specific modification sites [55].

Proteomics has revealed critical insights into pollutant-induced liver injury. For instance, PCBs alter mitochondrial protein profiles and disrupt energy metabolism through abnormal phosphorylation, Benzo[α]pyrene significantly reduced the activities of SOD3 and GPX, thereby disrupting the antioxidant system of hepatocytes and inducing liver injury [56]. Such findings provide essential molecular evidence for understanding pollutant-induced liver damage and identifying sensitive biomarkers for early detection and personalized toxicity evaluation. Abnormalities in key proteins and signaling pathways also suggest potential therapeutic targets for pollutant-associated liver diseases.

Metabolomics complements proteomics by investigating changes in metabolites, the end products of cellular processes. Pollutant exposure disrupts key metabolic pathways, including energy metabolism, oxidative stress, lipid metabolism, and amino acid metabolism. Metabolomics, using MS- or NMR-based techniques, enables high-resolution detection of metabolite concentrations and dynamic changes. For example, polycyclic aromatic hydrocarbons (PAHs) and heavy metals disrupt key metabolic pathways in hepatocytes, including the TCA cycle, β-oxidation, and bile acid metabolism. Specifically, PAHs inhibit the activity of enzymes like citrate synthase and isocitrate dehydrogenase, impairing the TCA cycle and reducing ATP production. Heavy metals, such as cadmium and lead, interfere with fatty acid β-oxidation by downregulating peroxisome proliferator-activated receptor alpha (PPARα) and carnitine palmitoyltransferase 1 (CPT1), leading to the accumulation of toxic lipid intermediates. Additionally, PAHs and heavy metals alter bile acid metabolism by suppressing farnesoid X receptor (FXR) signaling and upregulating cytochrome P450 7A1 (CYP7A1), resulting in the overproduction of toxic bile acids. These disruptions collectively lead to metabolic imbalances, oxidative stress, and hepatocyte dysfunction. In a chronic cadmium exposure model in mice, cadmium impairs the signaling of the gut microbiota bile acid intestinal farnesoid X receptor axis and increases hepatic bile acid (BA) synthesis, ultimately promoting the development of bile duct proliferation, inflammation, and liver damage [57]. The accumulation of oxidative stress markers (e.g., lipid peroxidation products and glutathione depletion) further implicates oxidative stress and lipid metabolism disorders as critical factors in pollutant-induced liver damage. Exposure to environmental pollutants induces changes in antioxidant enzymes, which serve as critical indicators of liver injury. For instance, SOD, a metalloenzyme with antioxidant properties, plays a pivotal role in neutralizing superoxide radicals, while GSH, a key reducing agent, is an essential component of the non-enzymatic antioxidant system. Following exposure to silica nanoparticles, the expression levels of both SOD and GSH in L-02 cells were significantly reduced, leading to cellular oxidative damage [58]. Furthermore, NADPH oxidase (NOX), an enzyme responsible for generating ROS, showed markedly increased protein levels of NOX1 and NOX2 after bisphenol S (BPS) exposure, resulting in an imbalance between oxidants and antioxidants and disrupting redox homeostasis [59]. In addition to enzymatic changes, metabolites can also mediate liver injury by disrupting cellular oxidative stress homeostasis. Metabolomics combined with lipidomics analysis revealed that Florfenicol (FF) induced significant alterations in lipids (toxic sphingolipid accumulation, excessive fatty acids, and acylglycerols), amino acids, TCA cycle intermediates, and nucleotides in zebrafish. These changes ultimately led to ATP depletion, elevated ROS, and oxidative stress, thereby promoting the progression of NAFLD [60]. Metabolomic analysis of perfluorinated alkyl substances (PFAS) revealed that they disrupt liver lipid metabolism by upregulating the expression of bile acids, triacylglycerols, and ceramides, leading to dysregulation of lipid metabolism-related pathways and contributing to the development of NAFLD [61]. Integrated proteomics and metabolomics approaches have provided comprehensive insights into pollutant-induced liver injury by linking altered proteins and metabolic pathways to toxicity mechanisms. These findings not only advance our understanding of pollutant toxicity but also offer novel strategies for biomarker discovery and therapeutic intervention.

Previous studies have extensively utilized multi-omics technologies in cancer research, such as proteomics investigations that have uncovered biomarkers and pathways associated with pancreatic cancer metastasis [62]. However, these studies primarily focus on pancreatic cancer, which significantly differs from our emphasis on liver disease progression. Other research has employed single-omics approaches to explore liver diseases, including lipidomics studies that identified metabolic pathways involved in the progression of non-alcoholic steatohepatitis (NASH) [63] and metabolomics analyses that revealed metabolic dysfunctions induced by hepatitis B virus (HBV) infection [64]. While these studies provide valuable insights, they are limited to single-omics approaches and do not encompass the broader context of liver disease progression or the impact of environmental pollutant exposure.

Several reviews have summarized the contributions of various omics technologies—such as epigenomics, transcriptomics, proteomics, metabolomics, and lipidomics—in understanding, diagnosing, and treating NAFLD [65]. Additionally, multi-omics analyses have offered insights into the mechanisms and biomarkers of metabolic dysfunction-associated steatotic liver disease (MASLD) [66]. However, these studies predominantly focus on individual liver diseases and do not address critical aspects such as immune toxicology or the broader progression of liver diseases.

In contrast, our study is the first to comprehensively address liver disease progression by integrating multiple dimensions, including cell crosstalk within the liver microenvironment, immune toxicology, and exposomics. This holistic approach not only identifies potential therapeutic targets but also provides a robust foundation for developing innovative diagnostic and treatment strategies. By bridging these gaps, we offer a novel perspective on liver disease progression, emphasizing the interplay between environmental exposures, immune responses, and cellular interactions within the liver.

## 4. Conclusions

By categorizing liver disease progression into stages of NAFLD, liver fibrosis, and HCC, this study explores the pathological mechanisms driving pollutant-induced inflammation–cancer transition. Specifically, we examine how pollutants, individually or synergistically, disrupt the liver microenvironment, cellular interactions, gene activation, and signaling pathways at different stages. Based on exposomics-related technologies and methods, we perform integrated analyses of genomic, transcriptomic, proteomic, and metabolomic data. For example, multi-omics research revealed that the risk score under TP53 mutation and senescence serves as a promising biomarker with the potential to aid in prognosis prediction. In the study of the effects of cytoplasmic polyadenylation element-binding protein 2 (CPEB2) on HCC, multi-omics analysis demonstrated that CPEB2 inhibits epithelial-to-mesenchymal transition (EMT) and metastasis of HCC through the regulation of the HIF-1α/miR-210-3p/CPEB2 axis. This finding suggests that targeting miR-210-3p may serve as a novel predictive biomarker and therapeutic strategy in HCC, enhancing the potential of CPEB2 expression. These analyses help uncover pollutant-induced genetic mutations, activation of inflammatory genes, protein modifications, and metabolic disturbances [67]. Our study provides insights into pollutant-induced liver damage mechanisms, paving the way for improved prevention, diagnosis, and personalized treatment of environment-related liver diseases while informing precision public health and environmental policy development.

## Data Availability

This is a review article and does not involve the generation or analysis of new experimental data, All data referenced in this article are sourced from previously published studies.
